# Supplementary material for: Defective plasticity in dermatomyositis patients muscle stem cells is associated with sustained intrinsic inflammatory signaling and disruption of the histone H3.3 chromatin loading pathway
Source: NAR Mol Med. 2026 May 23;3(2):ugag022. doi: 10.1093/narmme/ugag022 (PMC13199706; doi:10.1093/narmme/ugag022)
Supplement: ugag022_Supplemental_Files [file ugag022_supplemental_files.zip › 04 Legends Supplementary Figures V20.docx]

**Legends – Supplementary figures**

**Supplementary figure 1. Cell line description.** (**A**) Description of patients’ medical background, from which all DM-MuSCs used in this study were derived. (**B**) Description of patients’ medical background, from which HC-MuSCs were derived. Additional HC-MuSCs derived from healthy patientswere used in experiments involving only HC-MuSCs. (**C**) Comparison of the ages of HC versus DM patients. (**D**) Quantification of the percentage of CD56 positive cells after flow cytometry among the four HC-MuSC and four DM-MuSC described above.

**Supplementary figure 2. DM-MuSCs show profound transcriptomic defects.** (**A**) PCA of gene expression of HC- and DM-MuSCs in Prolif, DiffD1 and DiffD3 conditions. (**B**) Heatmap of the expression of DEGs between DM- and HC-MuSCs in Prolif, DiffD1 and DiffD3 conditions. On the X axis are samples sorted by culture condition and cell type. On the Y axis DEG set associated with genes up-regulated in DM-MuSCs and genes downregulated in HC-MuSCs. Overrepresented GO:Biological Process terms of each DEG set with a selection of representative genes are indicated in red for genes up-regulated in DM-MuSCs and in blue for genes downregulated in HC-MuSCs.

**Supplementary figure 3. DM-MuSCs show blunted plasticity in response to differentiation stimulus.** (**A)** to (**D**) Gene ontology biological process (GO:Biological process) over-representation analysis of the DEG between HC-MuSCs in Prolif *vs*. DiffD1 and between HC-MuSC at DiffD1 *vs*. DiffD3. (**A**) Over-represented terms in Prolif (Prolif *vs*. DiffD1). (**B**) Over-represented terms at DiffD1 (Prolif *vs*. DiffD1). (**C**) Over-represented terms at DiffD1 (DiffD1 *vs*. DiffD3). (**D**) Over-represented terms at DiffD3 (DiffD1 *vs*. DiffD3). (**E**) to (**H**) In the left panels, the mean expression of DEGs identified in Supplementary Fig. S2 (HC-MuSCs along the myogenesis process) was evaluated in both HC (green)- and DM (red)-MuSCs in the 3 conditions. The right panels show the expression in the RNA-seq data of selected genes belonging to the corresponding DEGs, with p-value significance between HC- and DM-MuSC indicated on the graph. (**E**) DEGs upregulated in Prolif (Prolif *vs*. DiffD1). Expression of *CXCL2*. (**F**) DEGs upregulated in DiffD1 (Prolif *vs*. DiffD1). Expression of *MYMX*. (**G**) DEGs upregulated in DiffD1 (DiffD1 *vs*. DiffD3). Expression of *CCNB1*. (**H**) DEGs upregulated in DiffD3 (DiffD1 *vs*. DiffD3). Expression of *MYH3*.

**Supplementary figure 4. Analysis of differentially expressed genes between HC-MuSCs in proliferation and differentiation.** (**A**) RT-qPCR of the log2FC expression of myogenic regulatory genes in Prolif in DM-MuSCs and HC-MuSCs, from four different patients each, normalized to their mean expression in HC-MuSCs. P-value significance between HC- and DM-MuSCs is indicated on the graph. (**B**) RT-qPCR of the log2FC expression of myogenic and cell-cycle regulatory genes in Prolif and DiffD3 DM-MuSCs and HC-MuSCs, from four different patients each, normalized to their mean expression in HC-MuSCs. P-value significance between HC- and DM-MuSCs is indicated on the graph. **(C**)-(**D**) Enrichment map of GO:Biological Process enriched in DEGs between HC- and DM-MuSCs at DiffD3. Cluster of similar terms are encircled and annotated. (**E**)-(**F**) EdU staining (green) of HC- and DM-MuSCs (nuclei blue) and quantification as percentage of positive cells in Prolif (**E**) and Diff3 (**F**) conditions. P-value significance between HC- and DM-MuSCs is indicated on the graph.

**Supplementary figure 5. Pro-inflammatory pathways activation in DM-MuSCs.** (**A**) Mean expression by cell type and by condition of all the genes involved in type I, II and III IFN signaling (blue panel), IL1 signaling (red panel), IL6 signaling (purple panel) and TNF-⍺ family signaling (orange panel) between DM- (red) and HC-MuSCs (green) in Prolif, DiffD1 and DiffD3 conditions. (**B**) Heatmap of the expression of genes coding for MHC, chemokines and T-cell receptor regulators in DM- and HC-MuSCs in Prolif, DiffD1 and DiffD3 conditions. (**C**) RT-qPCR of the log2FC expression of pro-inflammatory pathways target genes in Prolif in DM-MuSCs and HC-MuSCs, from four different patients each, normalized to their mean expression in HC-MuSCs. P-value significance between HC- and DM-MuSC is indicated on the graph.

**Supplementary figure 6. Activation of IFN signaling does not explain the defects observed in DM-MuSCs.** (**A**) Quantification of the mean percentage of EdU+ nuclei in HC- and DM-MuSCs from four different patients each, the experience was repeated on the same cells twice at different passages, in Prolif with or without addition of IFN-β (top panel). Quantification of the mean percentage of MYOG+ nuclei in HC- and DM-MuSC from three different patients each, the experience was repeated on the same cells twice at different passages, at DiffD3 with or without addition of IFN-β (bottom panel). (**B**) Expression of myogenic genes, non-myogenic control gene PDGFRA, IFN-stimulated genes and senescence-associated gene CDKN2A in Prolif in IFN-β treated HC-MuSCs from six different patients normalized to their mean expression in untreated HC-MuSCs (RT-qPCR). (**C**) Expression of myogenic genes and IFN-stimulated genes at DiffD3 in IFN-β treated HC-MuSCs from seven different patients normalized to their mean expression in untreated HC-MuSCs (RT-qPCR). (**D**) HC-MuSCs from seven different patients in differentiation at high density were treated or not with IFN-β and immunofluorescence for MYOG (green) and desmin (red) was performed (nuclei, blue). Representative fused myonuclei, MYOG+ mono-nucleated myocyte and MYOG- myoblast are zoomed. On the middle panel, quantification of the mean percentage of fused myonuclei and MYOG+ mono-nucleated myocytes between untreated and treated HC-MuSCs. On the right panel, quantification of the mean percentage of fused myonuclei between untreated and treated HC-MuSCs.

**Supplementary figure 7. TNF-⍺ stimulation induces the up-regulation of ISG expression.** Heatmap of the expression of 45 orthologs of ISGs (17) in mouse C2C12 cells treated or not with TNF-α in proliferation condition. Data re-analyzed from (30).

**Supplementary figure 8. Mid-term cytokine stimulation of HC-MuSCs does not phenocopy DM-MuSCs.** (**A**) Schematic of the mid-term cytokine treatment of HC-MuSCs. (**B**) Quantification of the mean percentage of EdU+ nuclei of HC-MuSCs from three different patients cultured for 8 days with or without IFN-β and/or TNF-α in the Prolif condition. (**C**) Quantification of the mean percentage of MYOG+ nuclei of HC-MuSCs from four different patients cultured for 9 days with or without IFN-β and/or TNF-α in DiffD3 condition. (**D**) Expression of myogenic genes, IFN- and TNF-⍺ stimulated genes of HC-MuSCs from five different patients cultured for 8 days with or without FN-β and/or TNF-α in the Prolif condition (RT-qPCR). (**E**) Expression of myogenic genes, IFN- and TNF-⍺ stimulated genes of HC-MuSCs from three different patients cultured for 9 days with or without IFN-β and/or TNF-α in the DiffD3 condition (RT-qPCR).

**Supplementary figure 9. H3.3 restoration partially rescues DM-MuSCs phenotype.** (**A**) Expression of *H3F3A* and *H3F3B* in DM-MuSCs and HC-MuSCs from 4 different patients each normalized to their mean expression in HC-MuSCs in the Prolif condition (RT-qPCR). (**B**) Western blot for H3.3 and desmin (DES) in HC- and DM-MuSCs from 4 different patients, the experience was repeated on the same cells twice at different passages, with the quantification of the signal of H3.3 normalized to DES for each sample. Blue crossbars indicate mean signal for each cell type. (**C**) Schematic of the strategy used to overexpress H3.3 in DM-MuSCs. DM-MuSCs were transduced with lentiviral vectors encoding either a doxycycline-inducible H3.3-HA construct or an empty vector as a control. After 48 h of puromycin selection, cells were seeded under either proliferation or differentiation conditions and cultured in the presence or absence of 100 ng/mL doxycycline. (**D**) Quantifications of the percentage of HA or EdU positive cells in stained proliferating DM-MuSCs transfected with a doxycycline-inducible H3.3-HA construct, with or without doxycycline. (**E**) Quantification of the mean percentage of EdU+ nuclei of HC-MuSCs transfected with either a control siLuc or double siRNA against H3F3A and H3F3B in the Prolif condition. (**F**) Quantification of the mean percentage of MYOG+ nuclei of of HC-MuSCs transfected with either a control siLuc or double siRNA against H3F3A and H3F3B in DiffD3 condition. (**G**) – (**H**) RT-qPCR of the log2FC of HC-MuSCs transfected with either a control siLuc or double siRNA against H3F3A and H3F3B in prolif (**G**) or DiffD3 (**H**). Delta Ct are normalized to their mean expression in cells transfected control siLuc. P-value significance between siLuc and double siH3.3 transfected cells is indicated on the graph.

**Supplementary figure 10. Inflammation driven loss of H3.3 is concentrated on MRFs and enhancers.** (**A**) Intensity plot on DEG up-regulated (left panel) or down-regulated (right panel) in DM-MuSCs in Prolif of the mean H3.3 signal in proliferating DM-MuSCs (in red) and HC-MuSCs (in green). (**B**) Intensity plot on DEG up-regulated (left panel) or down-regulated (right panel) in DM-MuSCs at DiffD3 of the mean H3.3 signal in DM-MuSCs (in dark red) and HC-MuSCs (in dark green) at DiffD3. For each panel in (**A**) and (**B**), differential mean H3.3 signal within the -5/+5kb of the TSS and TES of the selected genes was evaluated between HC- and DM-MuSCs, with each gene of the selected panel counted as a replicate. P-value significance between HC- and DM-MuSC is indicated on the graph. (**C**) Overlap of peaks with decreased H3.3 signal in DM-MuSCs, H3K4me1 peaks in HC-MuSCs (dataset from ENCODE) and H3K27ac peaks in HC-MuSCs (datasets from ENCODE, Zhang et al., 2020, <https://www.encodeproject.org/>).
