# Supplementary material for: Defective plasticity in dermatomyositis patients muscle stem cells is associated with sustained intrinsic inflammatory signaling and disruption of the histone H3.3 chromatin loading pathway
Source: NAR Mol Med. 2026 May 23;3(2):ugag022. doi: 10.1093/narmme/ugag022 (PMC13199706; doi:10.1093/narmme/ugag022)
Supplement: ugag022_Supplemental_Files [file ugag022_supplemental_files.zip › 07 Merged Supplementray Figures.pdf]

A. DM-MuSC line description

| Primary derived MuSCs name in this study | sDM1 | sDM3 | sDM5 | sDM6 |
|---|---|---|---|---|
| Patient number in Laure Gallay et al., 2022 Neurology | 4 | 6 | 7 | 8 |
| Age, years | 63 | 23 | 59 | 28 |
| Sex | M | F | M | F |
| Specific autoantibody | unknown | Mi2 | SAE2 | MDA5 |
| Diagnosis delay, months | 13 | 4 | 6 | 8 |
| Walking aid device requirement | yes | no | no | yes |
| Maximal creatine kinase, UI/L | 560 | 1200 | 6500 | 370 |
| Muscle biopsy site | Deltoid | Deltoid | Quadriceps | Deltoid |
| Histologic dermatomyositis severity score | 18 | 16 | 14 | 18 |

B. HC-MuSC line description

| Primary derived MuSCs name in this study | CT6 | CT7 | SK230 | SK236 |
|---|---|---|---|---|
| Patient number in Laure Gallay et al., 2022 Neurology | Na | Na | Na | Na |
| Age, years | 35 | 68 | 28 | 37 |
| Sex | M | F | F | M |
| Specific autoantibody | Na | Na | Na | Na |
| Diagnosis delay, months | Na | Na | Na | Na |
| Walking aid device requirement | Na | Na | Na | Na |
| Maximal creatine kinase, UI/L | Na | Na | Na | Na |
| Muscle biopsy site | | | | |
| Histologic dermatomyositis severity score | Na | Na | Na | Na |

C. Ages of patients

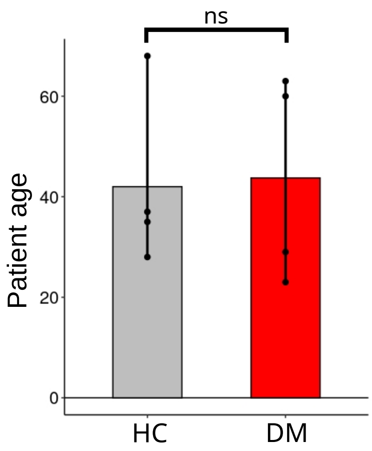


D. Percentage of CD56 positive cells in HC- and DM-MuSC

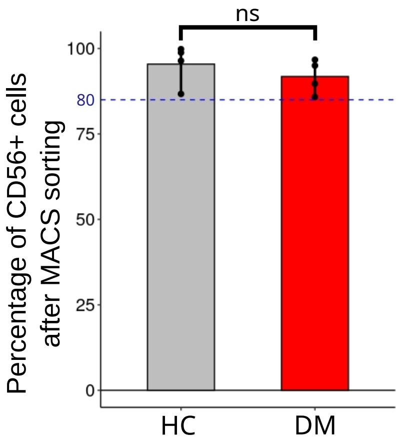

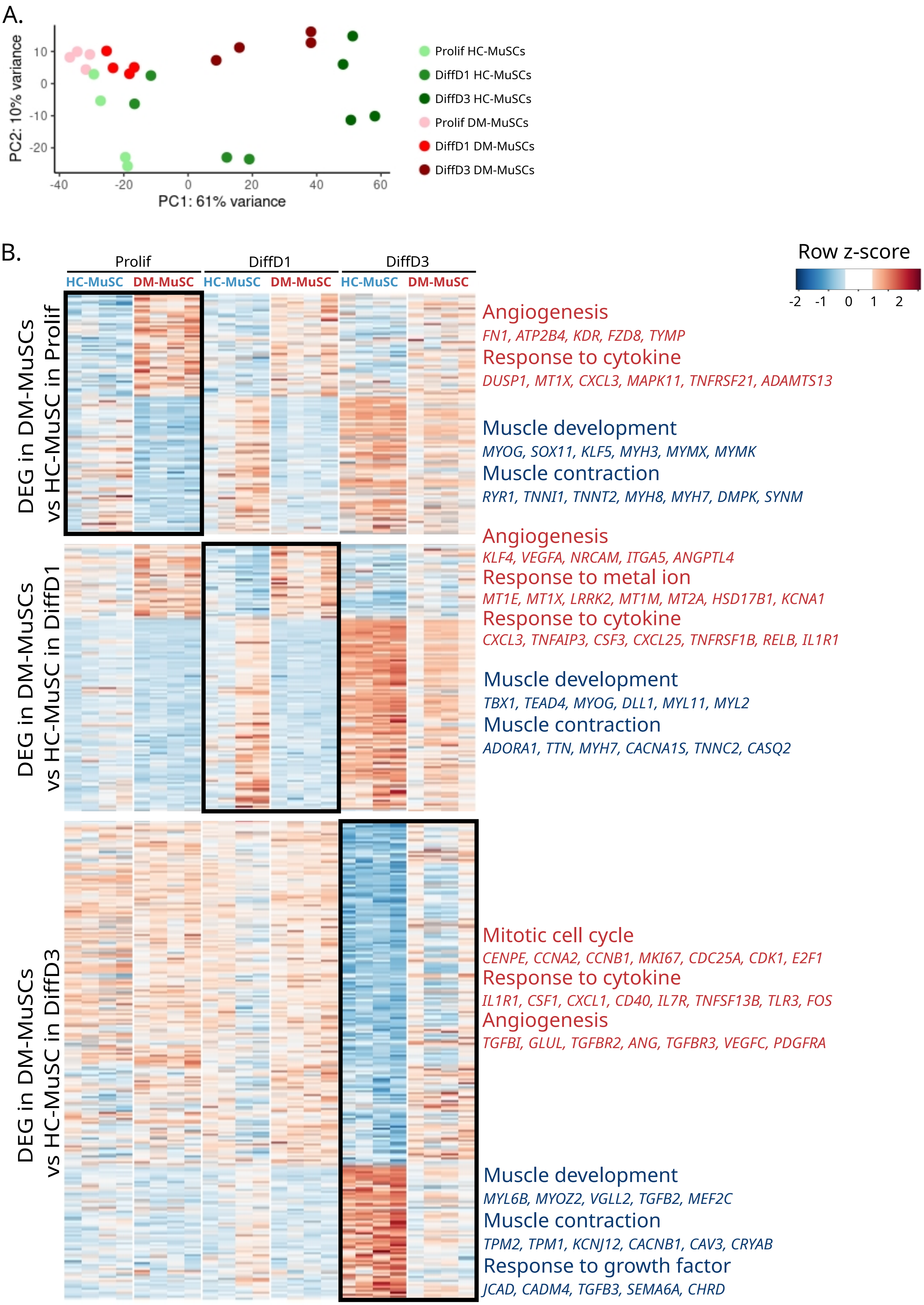

A.
PC2: 10% variance
10
0
-10
-20
-40
-20
0
20
40
60
PC1: 61% variance
Prolif HC-MuSCs
DiffD1 HC-MuSCs
DiffD3 HC-MuSCs
Prolif DM-MuSCs
DiffD1 DM-MuSCs
DiffD3 DM-MuSCs
B.
Prolif
DiffD1
DiffD3
HC-MuSC
DM-MuSC
HC-MuSC
DM-MuSC
HC-MuSC
DM-MuSC
Row z-score
-2
-1
0
1
2
DEG in DM-MuSCs vs HC-MuSC in Prolif
Angiogenesis
FN1, ATP2B4, KDR, FZD8, TYMP
Response to cytokine
DUSP1, MT1X, CXCL3, MAPK11, TNFRSF21, ADAMTS13
Muscle development
MYOG, SOX11, KLF5, MYH3, MYMX, MYMK
Muscle contraction
RYR1, TNNI1, TNNT2, MYH8, MYH7, DMPK, SYNM
DEG in DM-MuSCs vs HC-MuSC in DiffD1
Angiogenesis
KLF4, VEGFA, NRCAM, ITGA5, ANGPTL4
Response to metal ion
MT1E, MT1X, LRRK2, MT1M, MT2A, HSD17B1, KCNA1
Response to cytokine
CXCL3, TNFAIP3, CSF3, CXCL25, TNFRSF1B, RELB, IL1R1
Muscle development
TBX1, TEAD4, MYOG, DLL1, MYL11, MYL2
Muscle contraction
ADORA1, TTN, MYH7, CACNA1S, TNNC2, CASQ2
DEG in DM-MuSCs vs HC-MuSC in DiffD3
Mitotic cell cycle
CENPE, CCNA2, CCNB1, MKI67, CDC25A, CDK1, E2F1
Response to cytokine
IL1R1, CSF1, CXCL1, CD40, IL7R, TNFSF13B, TLR3, FOS
Angiogenesis
TGFBI, GLUL, TGFBR2, ANG, TGFBR3, VEGFC, PDGFRA
Muscle development
MYL6B, MYOZ2, VGLL2, TGFB2, MEF2C
Muscle contraction
TPM2, TPM1, KCNJ12, CACNB1, CAV3, CRYAB
Response to growth factor
JCAD, CADM4, TGFB3, SEMA6A, CHRD

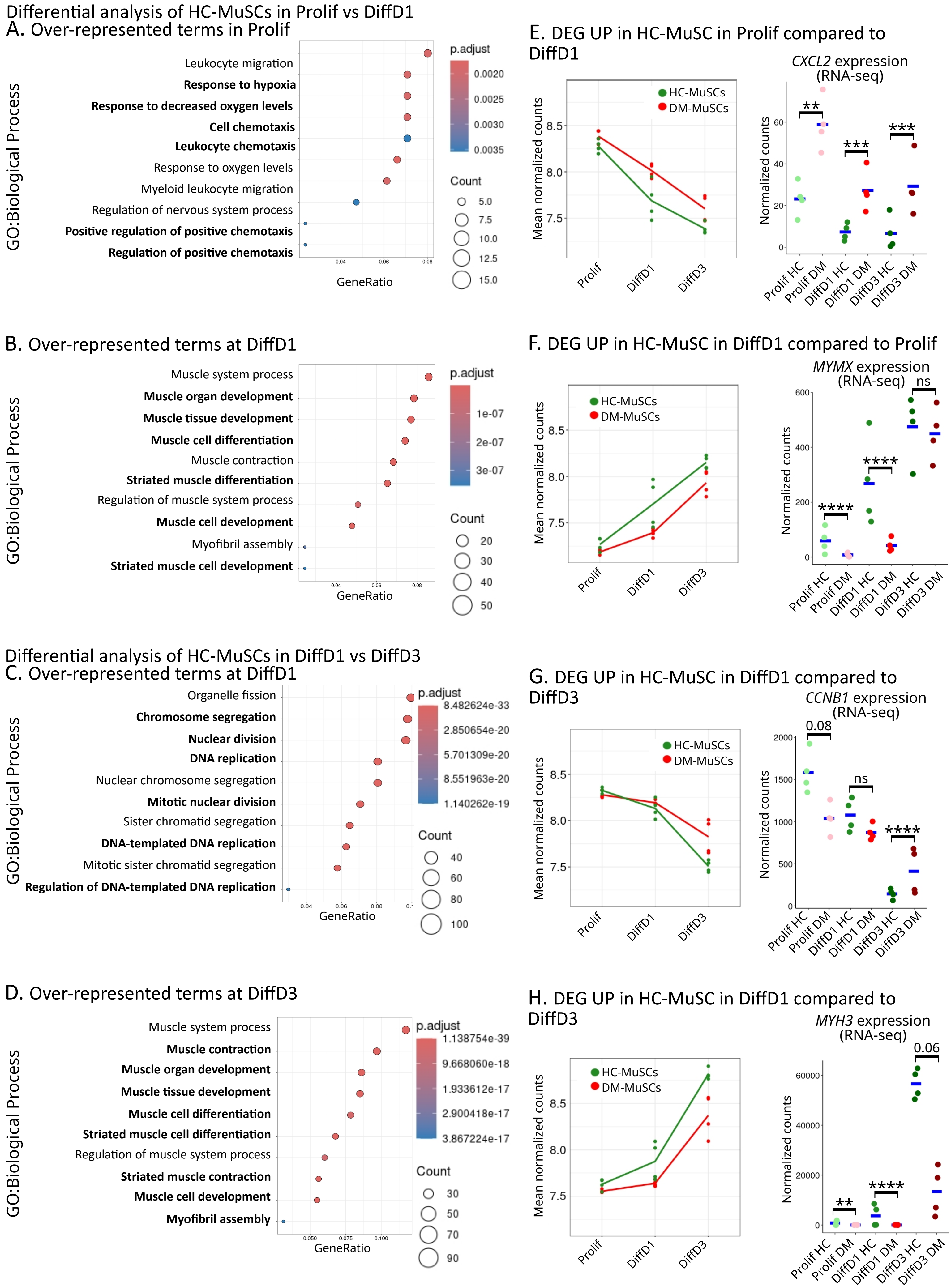
Differential analysis of HC-MuSCs in Prolif vs DiffD1
A. Over-represented terms in Prolif
GO:Biological Process
Leukocyte migration
Response to hypoxia
Response to decreased oxygen levels
Cell chemotaxis
Leukocyte chemotaxis
Response to oxygen levels
Myeloid leukocyte migration
Regulation of nervous system process
Positive regulation of positive chemotaxis
Regulation of positive chemotaxis
GeneRatio
p.adjust
0.0020
0.0025
0.0030
0.0035
Count
5.0
7.5
10.0
12.5
15.0
B. Over-represented terms at DiffD1
Muscle system process
Muscle organ development
Muscle tissue development
Muscle cell differentiation
Muscle contraction
Striated muscle differentiation
Regulation of muscle system process
Muscle cell development
Myofibril assembly
Striated muscle cell development
p.adjust
1e-07
2e-07
3e-07
Count
20
30
40
50
Differential analysis of HC-MuSCs in DiffD1 vs DiffD3
C. Over-represented terms at DiffD1
Organelle fission
Chromosome segregation
Nuclear division
DNA replication
Nuclear chromosome segregation
Mitotic nuclear division
Sister chromatid segregation
DNA-templated DNA replication
Mitotic sister chromatid segregation
Regulation of DNA-templated DNA replication
p.adjust
8.482624e-33
2.850654e-20
5.701309e-20
8.551963e-20
1.140262e-19
Count
40
60
80
100
D. Over-represented terms at DiffD3
Muscle system process
Muscle contraction
Muscle organ development
Muscle tissue development
Muscle cell differentiation
Striated muscle cell differentiation
Regulation of muscle system process
Striated muscle contraction
Muscle cell development
Myofibril assembly
p.adjust
1.138754e-39
9.668060e-18
1.933612e-17
2.900418e-17
3.867224e-17
Count
30
50
70
90
E. DEG UP in HC-MuSC in Prolif compared to DiffD1
HC-MuSCs
DM-MuSCs
Mean normalized counts
Prolif
DiffD1
DiffD3
CXCL2 expression (RNA-seq)
Normalized counts
Prolif HC
Prolif DM
DiffD1 HC
DiffD1 DM
DiffD3 HC
DiffD3 DM
F. DEG UP in HC-MuSC in DiffD1 compared to Prolif
MYMX expression (RNA-seq)
ns
G. DEG UP in HC-MuSC in DiffD1 compared to DiffD3
CCNB1 expression (RNA-seq)
0.08
ns
H. DEG UP in HC-MuSC in DiffD1 compared to DiffD3
MYH3 expression (RNA-seq)
0.06

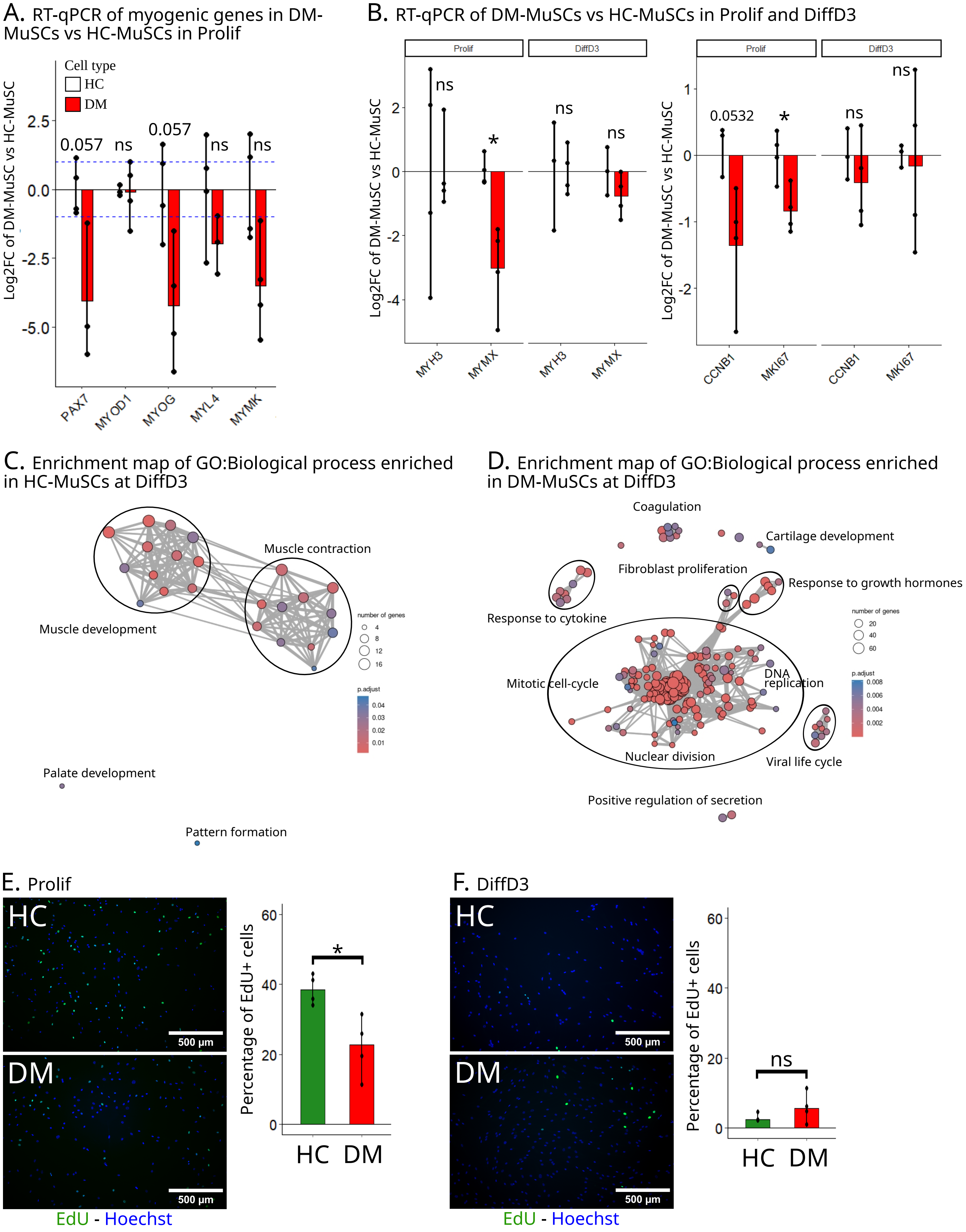

A. RT-qPCR of myogenic genes in DM-MuSCs vs HC-MuSCs in Prolif
Cell type
HC
DM
Log2FC of DM-MuSC vs HC-MuSC
0.057
ns
0.057
ns
ns
PAX7
MYOD1
MYOG
MYL4
MYMK
B. RT-qPCR of DM-MuSCs vs HC-MuSCs in Prolif and DiffD3
Prolif
DiffD3
ns
*
ns
ns
MYH3
MYMX
0.0532
*
ns
ns
CCNB1
MKI67
C. Enrichment map of GO:Biological process enriched in HC-MuSCs at DiffD3
Muscle contraction
Muscle development
Palate development
Pattern formation
number of genes
p.adjust
D. Enrichment map of GO:Biological process enriched in DM-MuSCs at DiffD3
Coagulation
Cartilage development
Fibroblast proliferation
Response to growth hormones
Response to cytokine
Mitotic cell-cycle
DNA replication
Nuclear division
Viral life cycle
Positive regulation of secretion
E. Prolif
HC
DM
500 µm
Percentage of EdU+ cells
*
EdU - Hoechst
F. DiffD3
HC
DM
500 µm
Percentage of EdU+ cells
ns
EdU - Hoechst

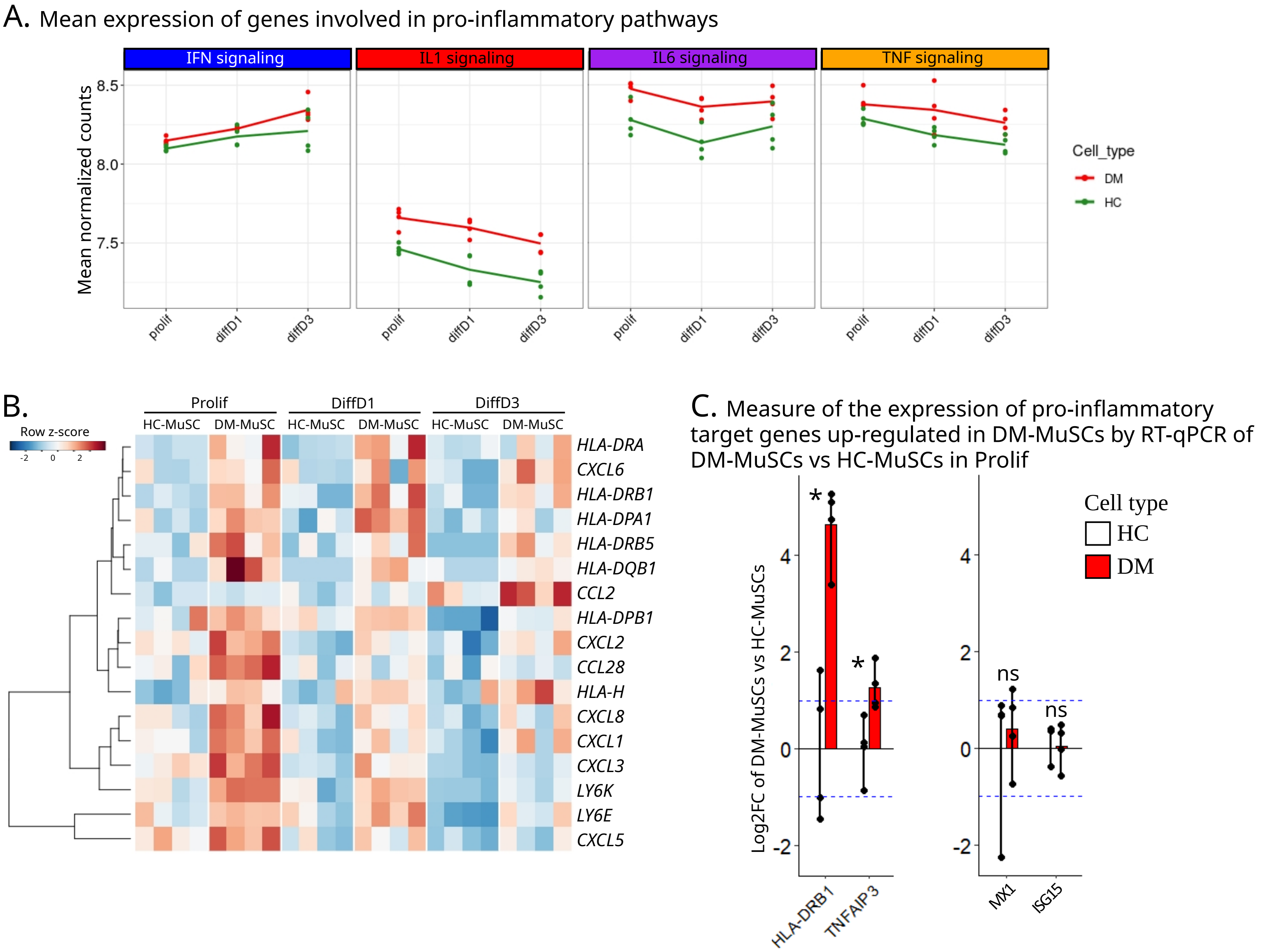
A. Mean expression of genes involved in pro-inflammatory pathways
IFN signaling
IL1 signaling
IL6 signaling
TNF signaling
Mean normalized counts
8.5
8.0
7.5
prolif
diffD1
diffD3
Cell_type
DM
HC
B.
Row z-score
-2
0
2
Prolif
DiffD1
DiffD3
HC-MuSC
DM-MuSC
HLA-DRA
CXCL6
HLA-DRB1
HLA-DPA1
HLA-DRB5
HLA-DQB1
CCL2
HLA-DPB1
CXCL2
CCL28
HLA-H
CXCL8
CXCL1
CXCL3
LY6K
LY6E
CXCL5
C. Measure of the expression of pro-inflammatory target genes up-regulated in DM-MuSCs by RT-qPCR of DM-MuSCs vs HC-MuSCs in Prolif
Log2FC of DM-MuSCs vs HC-MuSCs
4
2
0
-2
*
ns
HLA-DRB1
TNFAIP3
MX1
ISG15
Cell type
HC
DM

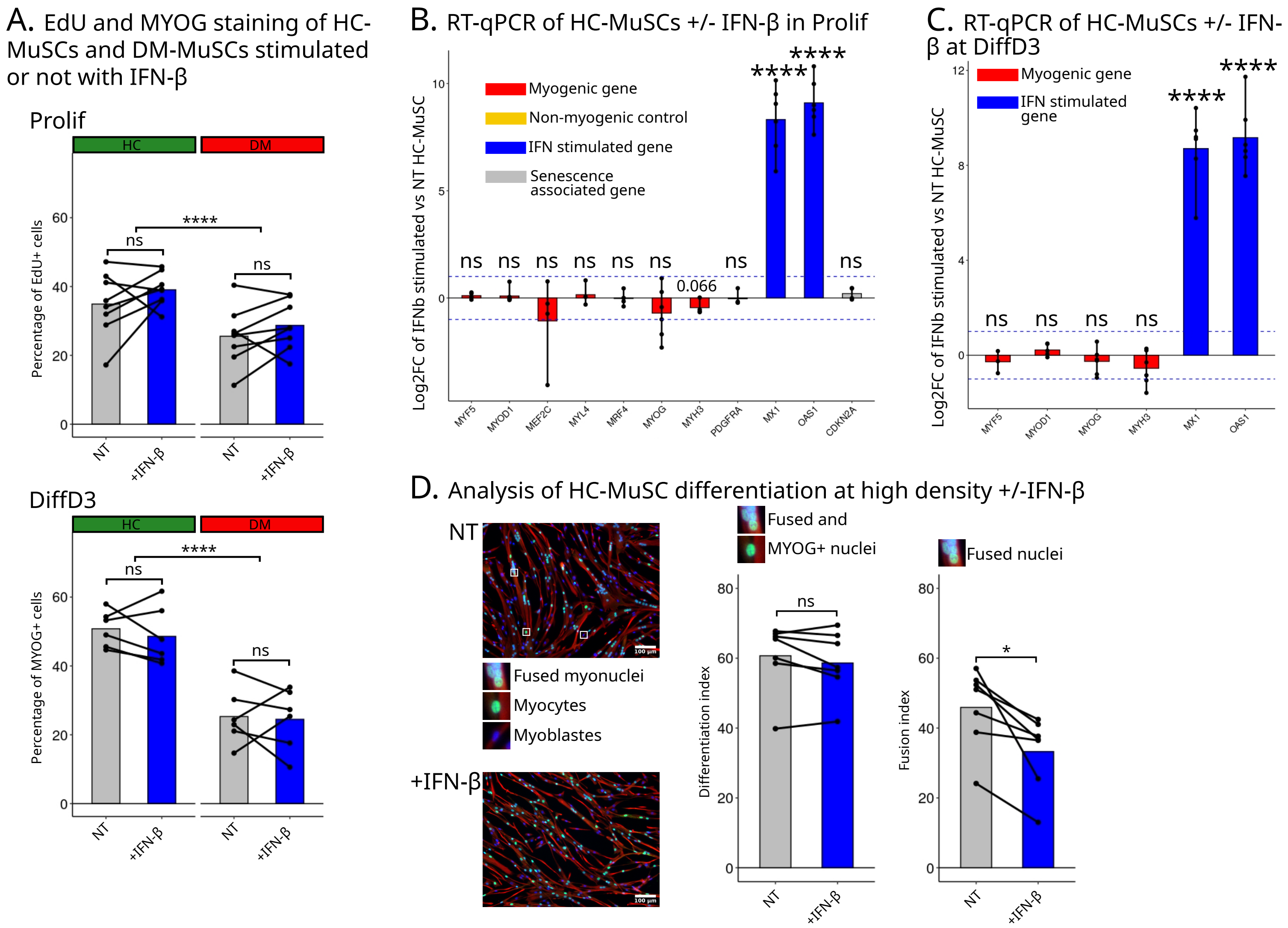
A. EdU and MYOG staining of HC-MuSCs and DM-MuSCs stimulated or not with IFN-β
Prolif
HC
DM
Percentage of EdU+ cells
ns
****
ns
NT
+IFN-β
DiffD3
Percentage of MYOG+ cells
B. RT-qPCR of HC-MuSCs +/- IFN-β in Prolif
Myogenic gene
Non-myogenic control
IFN stimulated gene
Senescence associated gene
Log2FC of IFNb stimulated vs NT HC-MuSC
0.066
MYF5
MYOD1
MEF2C
MYL4
MRF4
MYOG
MYH3
PDGFRA
MX1
OAS1
CDKN2A
C. RT-qPCR of HC-MuSCs +/- IFN-β at DiffD3
D. Analysis of HC-MuSC differentiation at high density +/-IFN-β
Fused myonuclei
Myocytes
Myoblastes
Fused and MYOG+ nuclei
Fused nuclei
Differentiation index
Fusion index
*

Expression of mouse orthologs of human ISGs in C2C12 stimulated with TNF-α

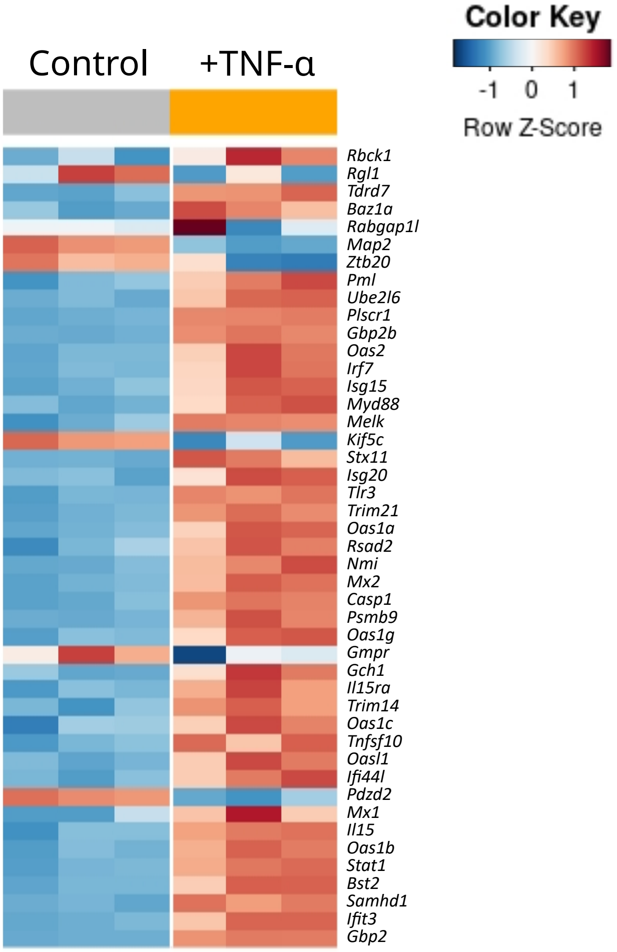

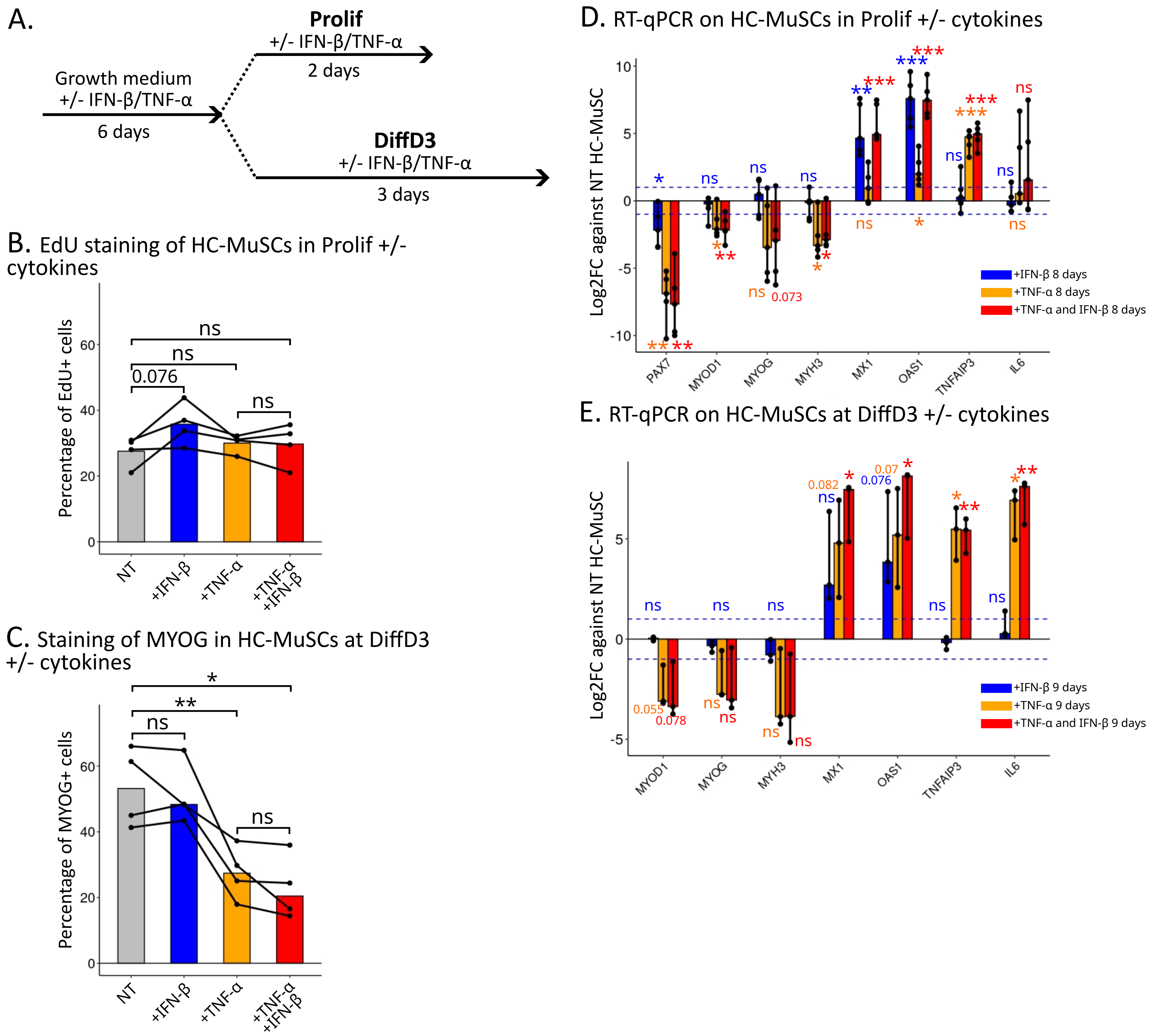

A.
Prolif
+/- IFN-β/TNF-α
2 days
Growth medium
+/- IFN-β/TNF-α
6 days
DiffD3
+/- IFN-β/TNF-α
3 days
B. EdU staining of HC-MuSCs in Prolif +/- cytokines
Percentage of EdU+ cells
0.076
ns
ns
ns
NT
+IFN-β
+TNF-α
+TNF-α +IFN-β
C. Staining of MYOG in HC-MuSCs at DiffD3 +/- cytokines
Percentage of MYOG+ cells
*
**
ns
ns
NT
+IFN-β
+TNF-α
+TNF-α +IFN-β
D. RT-qPCR on HC-MuSCs in Prolif +/- cytokines
Log2FC against NT HC-MuSC
PAX7
MYOD1
MYOG
MYH3
MX1
OAS1
TNFAIP3
IL6
0.073
+IFN-β 8 days
+TNF-α 8 days
+TNF-α and IFN-β 8 days
E. RT-qPCR on HC-MuSCs at DiffD3 +/- cytokines
Log2FC against NT HC-MuSC
MYOD1
MYOG
MYH3
MX1
OAS1
TNFAIP3
IL6
0.055
0.078
0.082
0.07
0.076
+IFN-β 9 days
+TNF-α 9 days
+TNF-α and IFN-β 9 days

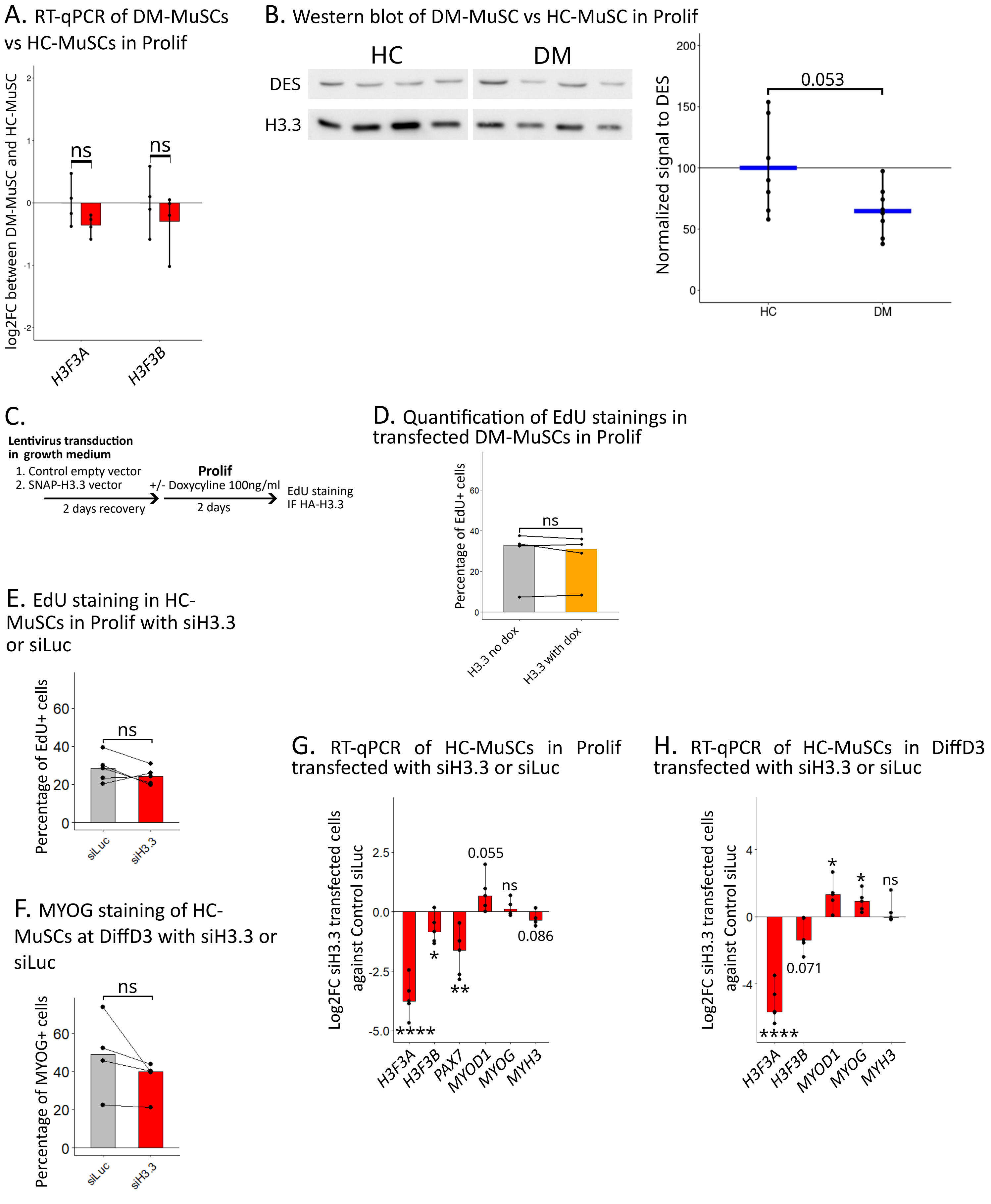

A. RT-qPCR of DM-MuSCs vs HC-MuSCs in Prolif
log2FC between DM-MuSC and HC-MuSC
ns
ns
H3F3A
H3F3B
B. Western blot of DM-MuSC vs HC-MuSC in Prolif
HC
DM
DES
H3.3
Normalized signal to DES
0.053
HC
DM
C.
Lentivirus transduction in growth medium
1. Control empty vector
2. SNAP-H3.3 vector
2 days recovery
Prolif
+/- Doxycyline 100ng/ml
2 days
EdU staining
IF HA-H3.3
D. Quantification of EdU stainings in transfected DM-MuSCs in Prolif
Percentage of EdU+ cells
ns
H3.3 no dox
H3.3 with dox
E. EdU staining in HC-MuSCs in Prolif with siH3.3 or siLuc
Percentage of EdU+ cells
ns
siLuc
siH3.3
F. MYOG staining of HC-MuSCs at DiffD3 with siH3.3 or siLuc
Percentage of MYOG+ cells
ns
siLuc
siH3.3
G. RT-qPCR of HC-MuSCs in Prolif transfected with siH3.3 or siLuc
Log2FC siH3.3 transfected cells against Control siLuc
0.055
ns
0.086
*
**
****
H3F3A
H3F3B
PAX7
MYOD1
MYOG
MYH3
H. RT-qPCR of HC-MuSCs in DiffD3 transfected with siH3.3 or siLuc
Log2FC siH3.3 transfected cells against Control siLuc
*
*
ns
0.071
****
H3F3A
H3F3B
MYOD1
MYOG
MYH3

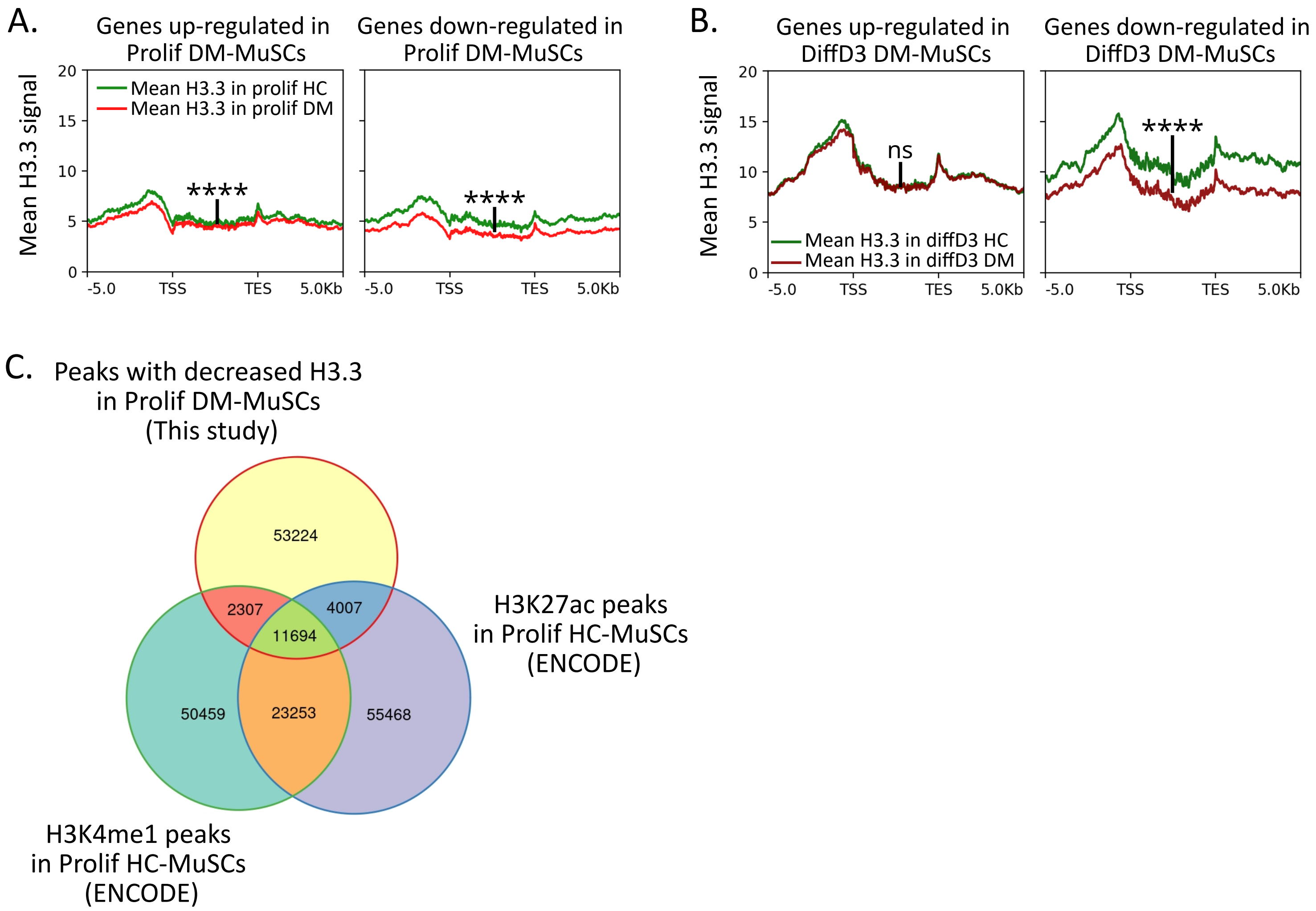

A.
Genes up-regulated in Prolif DM-MuSCs
Genes down-regulated in Prolif DM-MuSCs
Mean H3.3 signal
Mean H3.3 in prolif HC
Mean H3.3 in prolif DM
****
****
-5.0
TSS
TES
5.0Kb
B.
Genes up-regulated in DiffD3 DM-MuSCs
Genes down-regulated in DiffD3 DM-MuSCs
Mean H3.3 signal
ns
****
Mean H3.3 in diffD3 HC
Mean H3.3 in diffD3 DM
C.
Peaks with decreased H3.3 in Prolif DM-MuSCs (This study)
H3K27ac peaks in Prolif HC-MuSCs (ENCODE)
H3K4me1 peaks in Prolif HC-MuSCs (ENCODE)
53224
2307
4007
11694
50459
23253
55468
